# 3D slicer-based calculation of hematoma irregularity index for predicting hematoma expansion in intracerebral hemorrhage

**DOI:** 10.1186/s12883-022-02983-w

**Published:** 2022-12-05

**Authors:** Liping Cao, Meng Liu, Mengmeng Wang, Jian Ding, Keshi Mao, Kefeng Liu, Song Li

**Affiliations:** 1 Department of Neurology, The First People’s Hospital of Changzhou, The Third Affiliated Hospital of Soochow University, 213003 Changzhou, China; 2 Department of Neurosurgery, The Affiliated Changzhou No.2 People’s Hospital of Nanjing Medical University, No.29, Xinglong Lane, Jiangsu 213003 Changzhou, China

**Keywords:** Hematoma expansion, Intracerebral hemorrhage, Computed tomography, 3D slicer

## Abstract

**Background:**

Irregular hematoma is considered as a risk sign of hematoma expansion. The aim of this study was to quantify hematoma irregularity with computed tomography based on 3D Slicer.

**Methods:**

Patients with spontaneous intracerebral hemorrhage who underwent an initial and subsequent non-contrast computed tomography (CT) at a single medical center between January 2019 to January 2020 were retrospectively identified. The Digital Imaging and Communication in Medicine (DICOM) standard images were loaded into the 3D Slicer, and the surface area (S) and volume (V) of hematoma were calculated. The hematoma irregularity index (HII) was defined as $\frac{\sqrt{S/\pi}}2/\sqrt[3]{3V/4\pi}\times100$. Logistic regression analyses and receiver operating characteristic (ROC) curve analysis were performed to assess predictive performance of HII.

**Results:**

The enrolled patients were divided into those with hematoma enlargement (*n* = 36) and those without the enlargement (*n* = 57). HII in hematoma expansion group was 130.4 (125.1–140.0), and the index in non-enlarged hematoma group was 118.6 (113.5-122.3). There was significant difference in HII between the two groups (*P* < 0.01). Multivariate logistic regression analysis revealed that the HII was significantly associated with hematoma expansion before (odds ratio = 1.203, 95% confidence interval [CI], 1.115–1.298; *P* < 0.001) and after adjustment for age, hematoma volume, Glasgow Coma Scale score (odds ratio = 1.196, 95% CI, 1.102–1.298, *P* < 0.001). The area under the ROC curve was 0.86 (CI, 0.78–0.93, *P* < 0.01), and the best cutoff of HII for predicting hematoma growth was 123.8.

**Conclusion:**

As a quantitative indicator of irregular hematoma, HII can be calculated using the 3D Slicer. And the HII was independently correlated with hematoma expansion.

## Background

Spontaneous intracerebral hemorrhage (ICH) is the most sinister subtype of stroke, with a 1-month mortality rate exceeding 40%. And ICH affects approximately one million people each year worldwide [1, 2]. Hematoma expansion has been reported in 38% of patients with ICH and serves as an independent determinant of poor clinical outcome [3–5]. Hence, accurate identification of patients with a high risk of hematoma enlargement could facilitate the right clinical decisions.

The baseline computed tomography (CT) imaging is a convenient method for predicting hematoma evolution and enlargement [6, 7]. The parameters for predicting hematoma enlargement include larger baseline ICH volume, CT angiographic “spot sign”, clot density variation, island sign, CT blend sign, and lesion shape [8–12]. Recently, much attention has been paid to the influence of irregular shape on hematoma enlargement [13]. At present, quantitative approaches for assessing the irregularity of hematoma are still lacking. Therefore, development of a novel method for quantifying hematoma irregularity is crucial for early therapeutic intervention.

It is known that the surface area of the sphere is the smallest among objects with the same volume. Thus, the surface area of hematoma is increased with the increasing irregularity in the shape of hematoma. In the case of hematoma like a sphere, the radius is equal between the calculation using the surface area and that using the volume. Likewise, the ratio of the two radii is increased with the increasing irregularity in the shape of hematoma. In this study, we took the ratio of two radii as the hematoma irregularity index (HII). However, it remains to be solved how the surface area and volume of hematoma are accurately calculated. Currently, there are several methods available for measuring the volume of lesions, including Tada (ABC/2) formula hematomas [14], *Alice* [15], *MIStar* version 3.2 [16], *MIPAV* [17], *Leonardo* V [18], and *Analyze* 11.0 [19]. Among them, the Tada formula only provides a rough approximation of the volume of lesions because the measured object does not constitute an ellipse [16]. While the most software tools available for researchers are pricey and closed internal applications [20]. More recently, Chen et al. demonstrated that the 3D Slicer software is a stable and capable method for high-precision calculation of hematoma in comparison with slice method and voxelization method [21]. The 3D Slicer provides an efficient process for the measurement of lesion volume and surface area, while it has been used by a large and steadily growing user community in over 100 different research projects since 2003 [22]. Moreover, the 3D Slicer is an open source software platform and free for users. Thus, it has become an attractive candidate tool for medical image post-processing.

This study was intended to determine whether the HII can be obtained semi-automatically using the 3D Slicer. And the aim of our study was to investigate the value of the HII for predicting early hematoma expansion in patients with ICH.

## Methods

### Patients

This case series was approved by the Ethics Committees of Changzhou Second People’s Hospital Affiliated to Nanjing Medical University (Approval No: [2018]KY032-01).

This study retrospectively analyzed patients with primary ICH confirmed by CT, who were admitted to the neurosurgery department of Changzhou Second People’s Hospital between January 2019 and January 2020. Follow-up CT scans were performed within 24 h after the baseline CT scan. The exclusion criteria were as follows: (1) the radiological or clinical data were not available; (2) the hemorrhage was secondary to head trauma, neoplasms, vascular malformations, aneurysmal subarachnoid hemorrhage, or hemorrhagic transformation of brain infarction; (3) patients undergoing surgical evacuation of hematoma before the follow-up CT scan; and (4) those receiving an anticoagulant treatment. The baseline clinical variables were collected from the electronic medical record system, including age, gender, smoking history, alcohol consumption, hypertension, diabetes mellitus, interval between the onset of symptoms to baseline CT scanning, and Glasgow Coma Scale (GCS) score.

### Neuroimaging acquisition and analysis

CT-Scans were performed on a 16 slice Scanner (GE, American). Each head CT contains 28 slices of 5 mm thickness. As described previously [23], hematoma growth was defined as an increase of > 33% or an absolute increase of > 12.5 ml in hematoma volume as compared to the baseline ICH volume. The volume of intraventricular hematoma was excluded. The Digital Imaging and Communication in Medicine (DICOM) standard images were retrospectively obtained from our hospital’s Medicine radiology database. The hematoma volume and surface area were calculated independently by two raters who were blinded to the clinical information of patients. One month later, one of the raters repeated the above process.

### Calculation of HII based on the 3D slicer

**Fig. 1:**
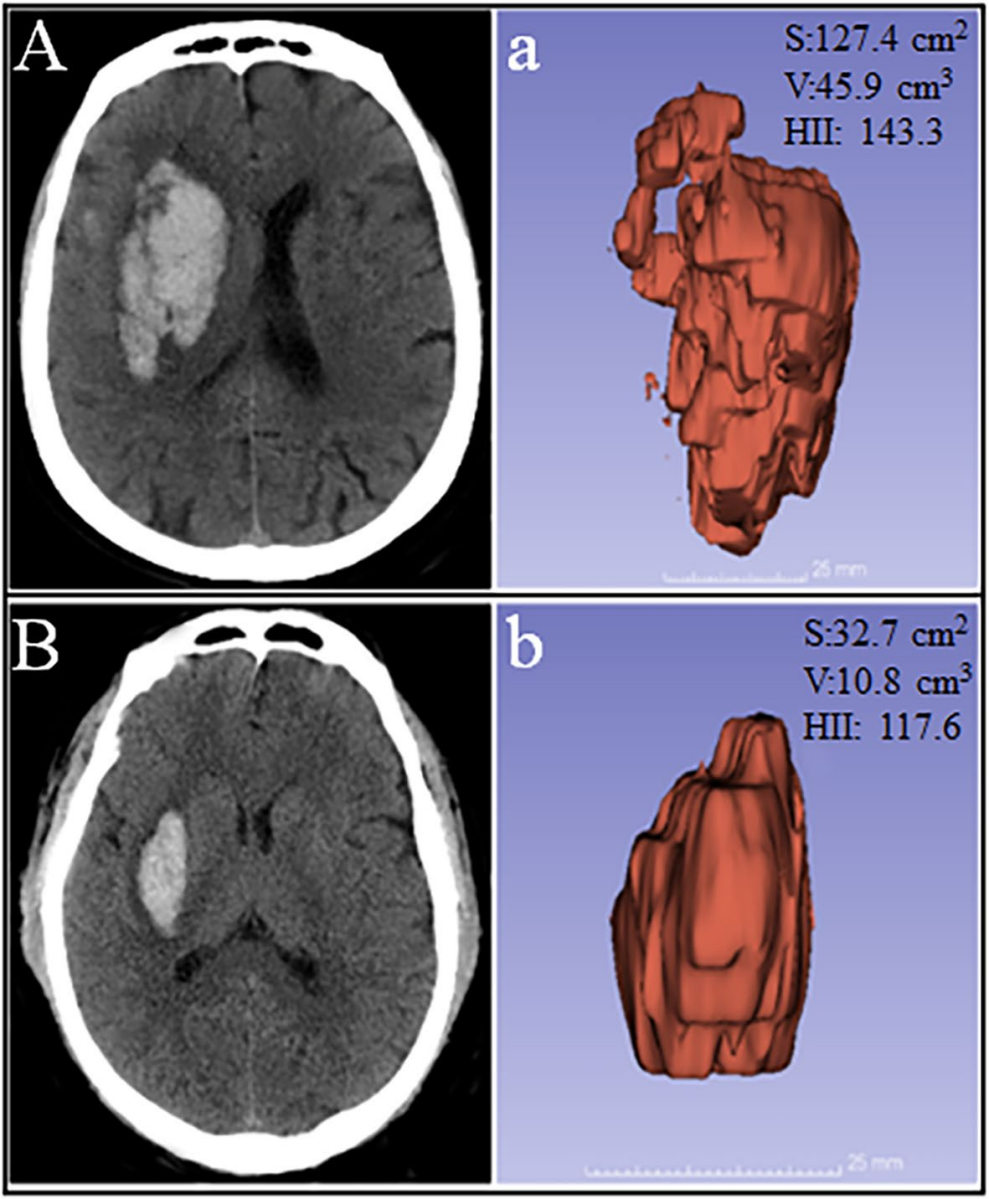
Hematoma was 3-dimensionally reconstructed with 3D Slicer from two ICH patients. (A and a) A example of a patient with hematoma expansion; (B and b) A example of patient without hematoma expansion; (A and B) original images; (a and b) The 3D model for hematoma. S, surface area; V, volume; HII, hematoma irregularity index

Three-dimensional reconstruction module in the 3D Slicer called Editor and Models (version 4.10.2) (www.slicer.org) was used for the calculation of HII. DICOM images data were transferred to a standard personal computer (Intel Core i5-9400 F CPU, 2.9 GHz, 16GB RAM) and then measured with *PaintEffect* in the *Editor* module. Hematomas were semi-automatically identified pixel by pixel in each slice, with thresholds ranging from 40 to 100 HU [24]. Finally, the *Models* module was utilized to reconstruct the three-dimensional data by adding up all the pixels from each slice, and data on volumes (V) and surface area (S) were directly obtained from the 3D Slicer (Fig. 1). Both the baseline CT scan and the second scan were performed to calculate the hematoma volume using the same method and criteria as above.

**Fig. 2:**
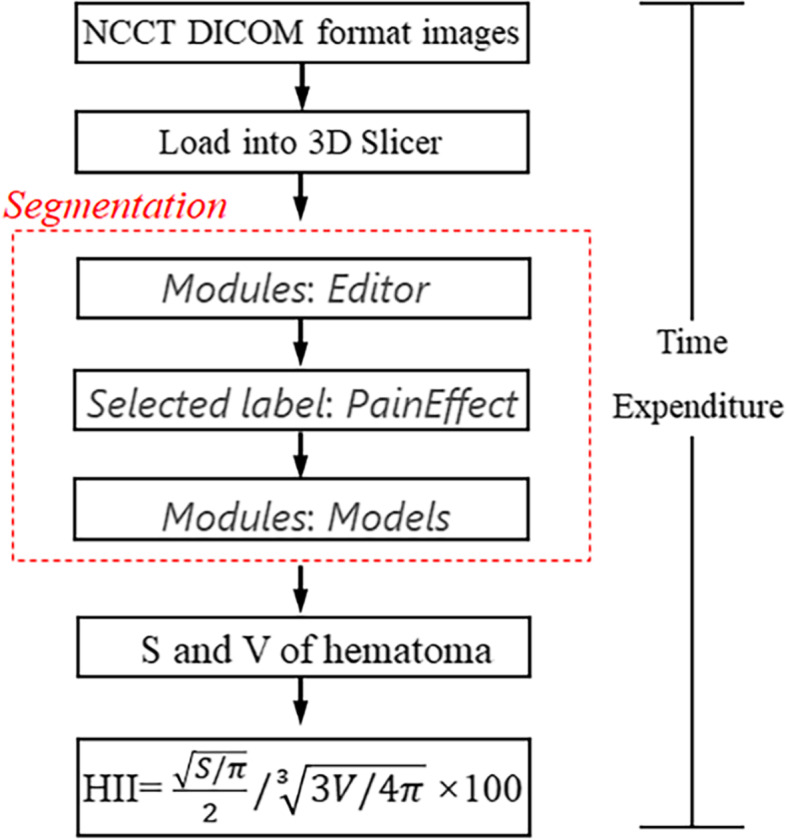
Detailed workflow for the calculation of HII. DICOM images data were transferred to 3D Slicer, and hematomas were semi-automatically identified pixel by pixel in each slice with *PaintEffect* in* Editor* module. The *Models* module in the software was utilized to reconstruct a three-dimensional data by adding up volume of all the pixels, and data on the volumes and surface area were directly obtained from the software. The hematoma irregularity index was defined as  $\sqrt{\frac{\mathrm S/\mathrm\pi}2}/\sqrt[3]{3V/4\mathrm\pi}{}^\ast100$. The time required for the entire process was recorded. S, surface area; V, volume; HII, hematoma irregularity index

Additionally, the R_1_ of a sphere with the S and R_2_ of a sphere with the V were calculated. The HII was defined as R_1_/R_2_ × 100 according to the following formula (Fig. 2).

$${\mathrm R}_1=\frac{\sqrt{\displaystyle S/\pi}}2,\;{\mathrm R}_2=\sqrt[3]{3V/4\pi};\;\;\;\;\;\;\;\;\;\;\;\mathrm{HII}={\mathrm R}_1/{\mathrm R}_2\times100$$

### Time expenditure

The images were measured three times, totaling 279 measurements for 93 images. The interval from starting tag to data acquisition was recorded using a stopwatch in every measurement.

### Statistical analysis

Statistical analyses were performed using the SPSS software (Version 22.0, Chicago, IL, USA). The Shapiro-Wilk test was conducted to determine whether or not the data were distributed normally. While continuous variables with a normal distribution were expressed as mean ± SD, those with a non-normal distribution were expressed as median (interquartile range). The unpaired *t*-test and Mann–Whitney’s *U* test were performed to compare 10 variables. Intra- and inter-rater reliability were examined with intraclass correlation coefficients (ICCs) using 1-way analysis of variance. Univariate and multivariate logistic regressions were used to calculate the odds ratio and corresponding 95% confidence interval (CI) for identifying independent risk factors in hematoma expansion. Receiver operating characteristic (ROC) curve analysis was carried out to assess predictive performance. *P* < 0.05 indicated statistical significance.

## Results

### Baseline clinical data

**Fig. 3:**
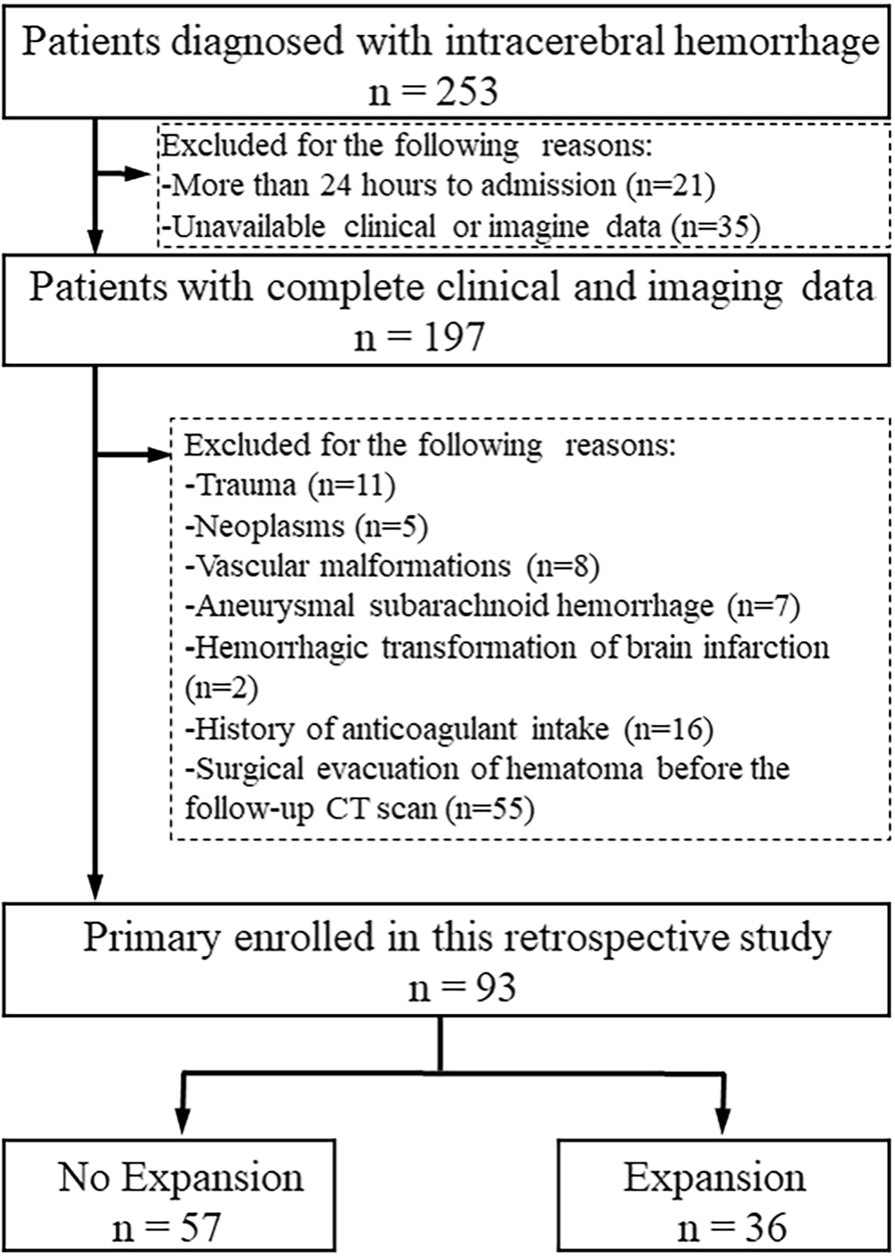
Flowchart of patient enrollment

A total of 93 patients met the inclusion criteria and were enrolled in the study. The enrollment was illustrated in the flowchart (Fig. 3). Among the patients (mean age, 64.2 ± 14.0 years), 41 (44.1%) were female. Median (interquartile range [IQR]) interval from the onset of symptoms to the baseline CT scanning was 3.0 (2.0–5.0) hours, and median (IQR) Glasgow Coma Scale (GCS) score at presentation was 13 (10–15). Besides, 72 (77.4%), 7 (7.5%), 17 (18.3%), and 17 (18.3%) out of the 93 patients had hypertension, diabetes mellitus, alcohol consumption, and smoking history, respectively. Median (IQR) baseline ICH volume was 10.8 (4.1–20.6) ml, and median (IQR) HII was 121.4 (116.7-130.1).

**Fig. 4:**
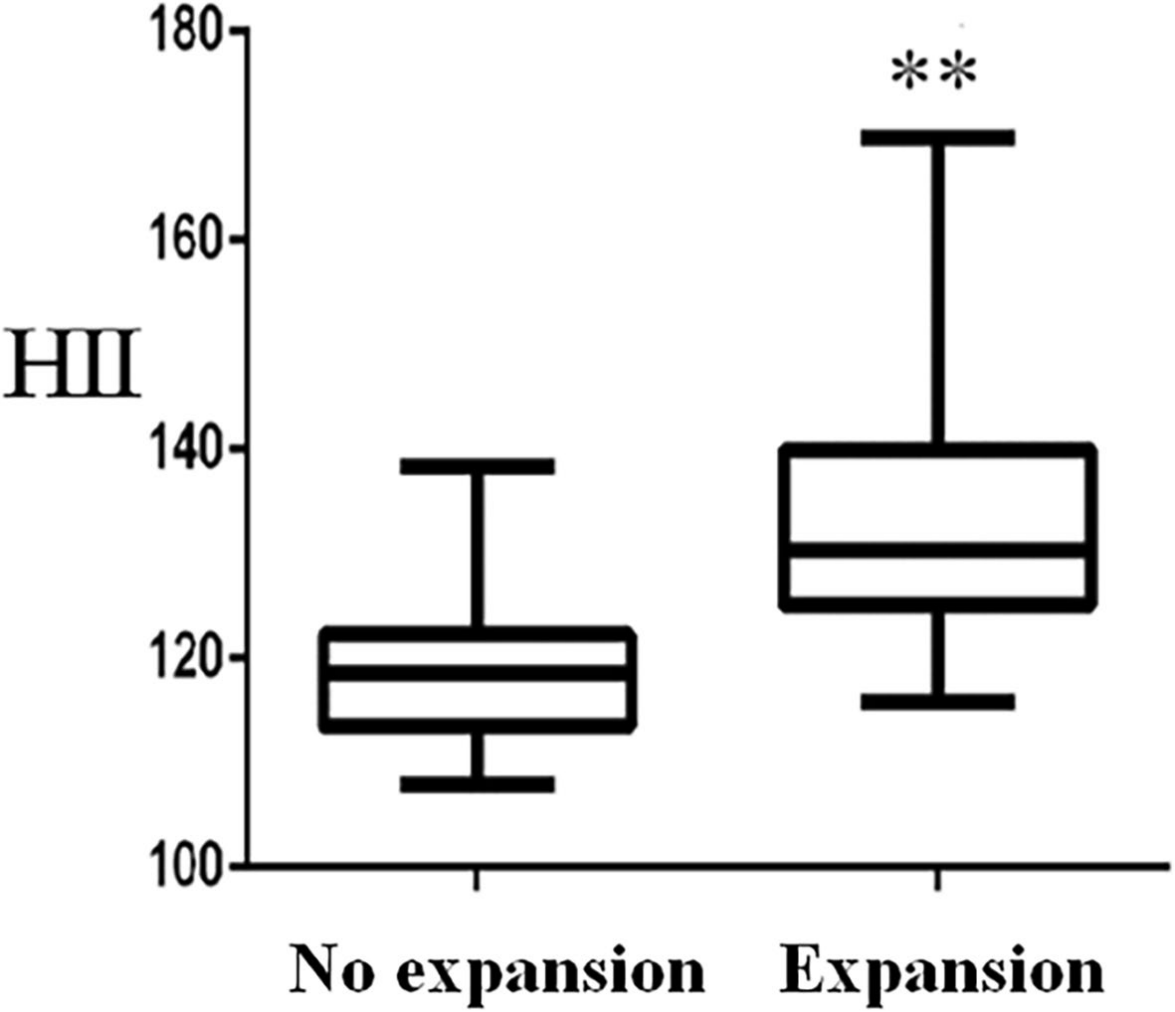
Comparison of HII between different groups using Mann-Whitney U test. The box plot presents the upper and lower quartiles, median, and extremums. ** *P <* 0.01 versus no expansion group. HII, hematoma irregularity index

As shown in Table 1, there were no significant differences in age, gender, alcohol consumption, smoking, hypertension, diabetes mellitus and interval between the onset of symptoms to baseline CT scanning between the two groups (all *P* > 0.05). The HII in patients with hematoma expansion (130.4, 125.1–140.0) was higher than that in those without hematoma expansion (118.6, 113.5-122.3) (*P* < 0.01, Fig. 4). Patients with hematoma expansion have a larger baseline ICH volume and lower GCS score compared to those without hematoma expansion (*P* < 0.01 for both).

**Table 1:** Comparison of baseline demographic, clinical, and radiological characteristics between patients with and without hematoma expansion

Variable	Hematoma expansion (*n* = 36)	No expansion (*n* = 57)	*p*-value
Age, mean (SD), y	67.5 (13.5)	62.1 (14.0)	0.07
Female sex, No. (%)	14 (38.9)	27 (47.4)	0.42
Alcohol consumption, No. (%)	6 (16.7)	11 (19.3)	0.75
Smoking, No. (%)	8 (22.2)	9 (15.8)	0.43
Hypertension, No. (%)	28 (77.8)	44 (77.2)	0.95
Diabetes mellitus, No. (%)	1 (2.8)	6 (10.5)	0.24
Hematoma volume, median (IQR), mL	16.5 (11.7–22.1)	6.6 (2.7–11.3)	< 0.01
Glasgow Coma Scale score, median (IQR)	11.5 (9.3–14)	14 (11.5–15)	< 0.01
Time to baseline CT, median (IQR), hours	2.0 (2.0–3.0)	3.0 (2.0–8.0)	0.12
HII median (IQR)	130.4 (125.1–140.0)	118.6(113.5-122.3)	< 0.01

*CT *Computed tomography, *SD *Standard deviation, *IQR *Interquartile range, *HII *Hematoma irregularity index

### HII measurements

The HII and ICCs are summarized in Table 2. A HII of 121.34 (115.51-130.92) and 123.32 (116.44-131.02) was presented by the two raters. There was no significant difference in HII between two raters (*P* = 0.227). HII obtained from the re-test of rater1 was 122.51 (116.41–131.30), and no significant differences were found among intra-rater measurements (*P* = 0.199). The inter-rater ICC in this test was 0.936 (*P* < 0.001), and intra-rater ICC was 0.956 ( *P* < 0.001). The above data indicated that there is an excellent agreement in HII between raters. Median interval from starting tag to data acquisition was 67.0 (60.0–74.0) seconds for the 279 measurements.

**Table 2:** Summary of HII Measurements

	HII median (IQR)	Wilcoxon Rank-Sum *P* Value	Inter-rater ICC
Rater 1	121.34 (115.51-130.92)		
Rater 2	123.32 (116.44-131.02)		
		0.227	0.936 (< 0.001)
Re-test of rater 1	122.51(116.41–131.30)	0.199	0.956 (< 0.001)

*IQR *Interquartile range, *ICC *Intraclass correlation coefficient, *HII *Hematoma irregularity index

## Prediction of hematoma expansion

Univariate logistic analysis revealed that the odds ratio (OR) of age, initial hematoma volume, GCS score, and HII were 1.029 (95% CI, 0.997–1.062, *P* = 0.072), 1.042 (95% CI, 1.011–1.074, *P* = 0.008), 0.842 (95% CI, 0.726–0.976, *P* = 0.023), and 1.203 (95% CI, 1.115–1.298, *P* < 0.001), respectively. Variables with *P* < 0.1 in univariate logistic analysis were included into the multivariate logistic regression model. After adjustment for confounders (age, hematoma volume, GCS score), HII was an independent risk factor for hematoma expansion (OR, 1.196 95% CI, 1.102–1.298; *P* < 0.001) (Table 3). Moreover, Hosmer and Lemeshow goodness-of-fit test showed that the model fits the data well (*P* = 0.597).

**Table 3:** Crude odds ratio and adjusted odds ratio according to logistic regression analysis

Variable	Crude OR	95% CI	Adjusted OR (enter)	95% CI	*P*-value
Age	1.029	0.997–1.062	1.017	0.977–1.059	0.415
Hematoma volume	1.042	1.011–1.074	0.992	0.950–1.036	0.726
Glasgow Coma Scale score	0.842	0.726–0.976	1.002	0.813–1.237	0.982
HII	1.203	1.115–1.298	1.196	1.102–1.298	< 0.001

*CI *Confidence interval, *HII *Hematoma irregularity index, *OR *Odds ratio

**Fig. 5:**
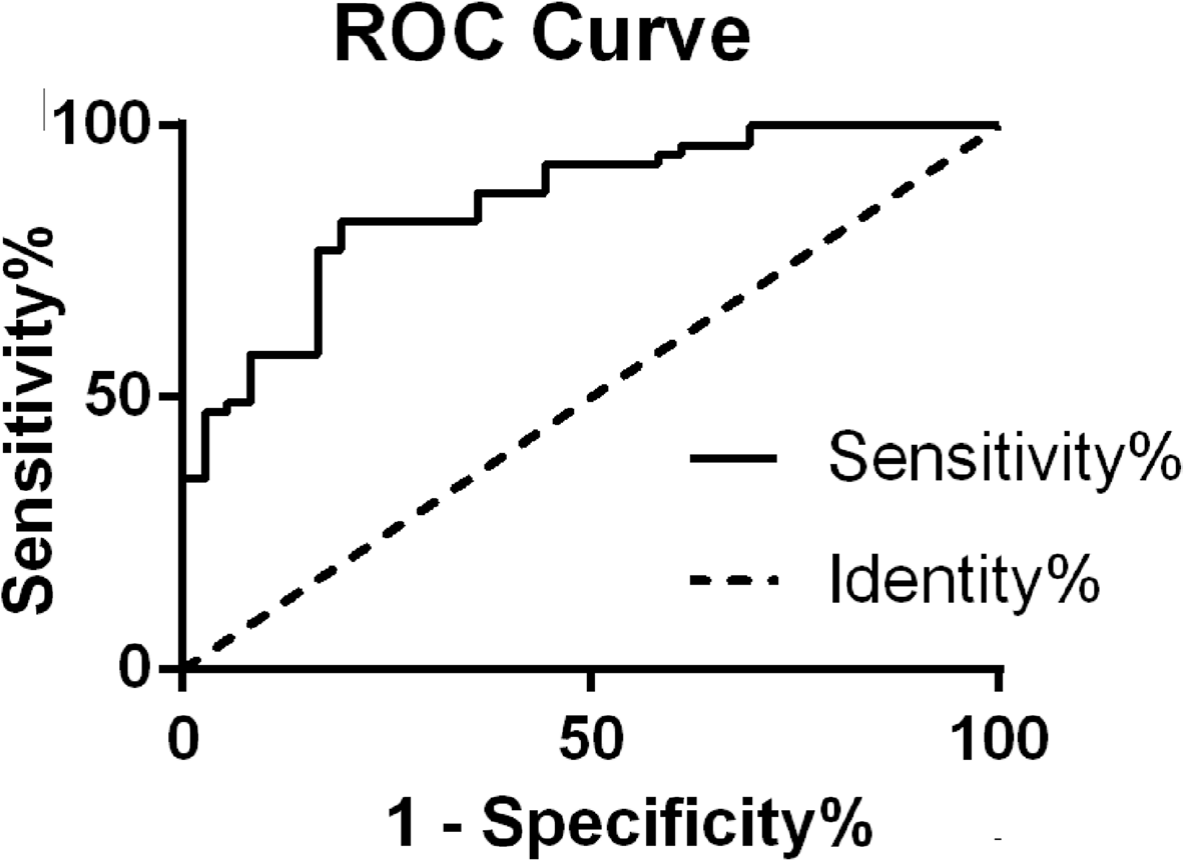
Receiver operating characteristic curve analysis. ROC curve analysis using a binary definition of hematoma expansion revealed that an area under the curve was 0.86 (*P* < 0.01), and the best cutoff of HII for predicting hematoma growth was 123.8% (sensitivity 81%, specificity 83%). HII, hematoma irregularity index. ROC, receiver operating characteristic

The ROC curve demonstrated that HII has potential for predicting hematoma expansion (Fig. 5). The area under the curve (AUC) was 0.86 (CI, 0.78–0.93, *P* < 0.01). According to the method of Youden [25], the best cutoff of HII for predicting hematoma growth was 123.8 (sensitivity, 81%; specificity, 83%).

## Discussion

In this study, we identified HII as a reliable indicator for quantifying hematoma irregularity based on CT images. The HII can be semi-automatically obtained with the 3D Slicer. We further showed that HII in the hematoma expansion group was higher than that in the non-enlarged hematoma group, while the HII was independently correlated with hematoma expansion.

Consistent with the previous studies [26, 27], we demonstrated that the 3D Slicer can be successfully used for modelling the hematoma (Fig. 1). Xu et al. reconstructed the hematoma using the 3D Slicer and showed that the Tada formula was inadequate in calculating the volume of irregular hematomas [26]. Strik et al. reported that volumetric data obtained from the 3D Slicer tended to be better correlated with the clinical course than the distance of midline shift and volume of hemorrhage from the conventional assessment [27]. It has also been demonstrated that the reconstruction of hematoma based on the 3D Slicer can be used in hematoma removal surgery [28]. Altogether, these findings indicate that 3D Slicer-based hematoma reconstruction is a well-established approach.

While the *Editor* module in the 3D Slicer offers a convenient method for delineating hematoma areas, the *Models* module in the software provides data on the volume and surface area of hematoma. The 3D Slicer has been used to calculate the surface area and volume of the region of interest [29, 30]. In this study, we redefined HII by using S and V. The HII is measured based on the theory that the surface area of the sphere is smallest among objects with the same volume. In the present study, the R1 of a sphere with the S and R2 of a sphere with the V were calculated, and the HII was then defined as V1/V2 × 100 (Fig. 2). Herein, we provided the first demonstration that the irregularity of hematoma can be calculated by the above formula. Furthermore, we showed that the semi-automatic calculation method in the 3D Slicer ensures fast and accurate data acquisition. Recently, Zhao et al. took 3D Slicer-based measurement of hematoma volume as the gold standard to evaluate the accuracy of the formula method [24]. In the meantime, it has been reported that 3D Slicer-based segmentation of subcutaneous xenograft tumor (ICC = 0.9999) resulted in higher reliability than the manual segmentation (ICC = 0.9996–0.9998) [31]. Similarly, we found that the inter-rater ICC in this test was 0.936 and intra-rater ICC was 0.956, indicating that there was good reliability for the grading scale between the raters. In addition, we observed that the calculation process only took 67.0 (60.0–74.0) seconds, showing that it is a time-saving process. Consistently, a study on reconstructing glioblastoma revealed that the time required for semi-automatic segmentation with 3D Slicer was on an average 61% of the time required for a pure manual segmentation [32].

The present study showed that HII was elevated in the expansion group as compared to the non-expansion group. To determine whether the HII indicates an enlargement of hematoma, we performed multivariate logistic regression analysis and identified HII as an independent risk factor for hematoma expansion (OR 1.196, 95% CI, 1.102–1.298; *P* < 0.01). Consistent with the previous studies [12, 33], we found that patients with hematoma expansion were more likely to have larger ICH volumes as well as lower GCS scores. Recently, intensive SBP lowering reduced the frequency of hematoma expansion in patients with moderate to severe grade ICH [34]. Meantime, ultra-early blood pressure reduction could decrease hematoma growth and improves outcome in intracerebral hemorrhage [35]. In our study, all patients included had their blood pressure controlled according to the same clinical guidelines. Based on this, it is concluded that HII predicts hematoma expansion. Therefore, management of blood pressure should not be overlooked because of low HII.

The current study has several potential limitations. First, this study excluded patients who had undergone surgical evacuation of hematoma before the follow-up CT scan. This may reduce the proportion of patients with hematoma expansion. Second, this was a retrospective single-center study, and the relatively small sample size could limit statistical power of the model. Therefore, further studies with a larger sample size at multiple medical facilities are required for verifying the results in this study. Third, the correlation between HII and long-term clinic functional outcomes worthy of verification through larger sample trials of long time follow-up.

## Conclusion

In this study, we developed a reliable method for semi-automatic quantification of hematoma irregularity following ICH based on CT images. We showed that while the HII was dependently correlated with hematoma expansion, the best cutoff of HII for predicting hematoma growth was 123.8. Hence, the present study provides a novel strategy for predicting hematoma expansion after intracerebral hemorrhage.

## Data Availability

The datasets used and/or analysed during the current study are available from the corresponding author on reasonable request.

## Abbreviations

HII: Hematoma irregularity index
ROC: Receiver operating characteristic
CI: Confidence interval
CT: Computed tomography
ICH: Intracerebral hemorrhage
GCS: Glasgow Coma Scale
IQR: Interquartile range
OR: Odds ratio
AUC: Area under the curve
